# Continuous braided suturing technique for robotic mitral valve annuloplasty

**DOI:** 10.1016/j.xjtc.2026.102321

**Published:** 2026-03-20

**Authors:** Norihiko Ishikawa, Go Watanabe, Daiki Yoshiyama, Toru Koakutsu, Takafumi Horikawa, Ryuta Seguchi, Shigeyuki Tomita, Toshiya Ohtsuka

**Affiliations:** Department of Cardiovascular Surgery, NewHeart Watanabe Institute, Tokyo, Japan

**Figure:**
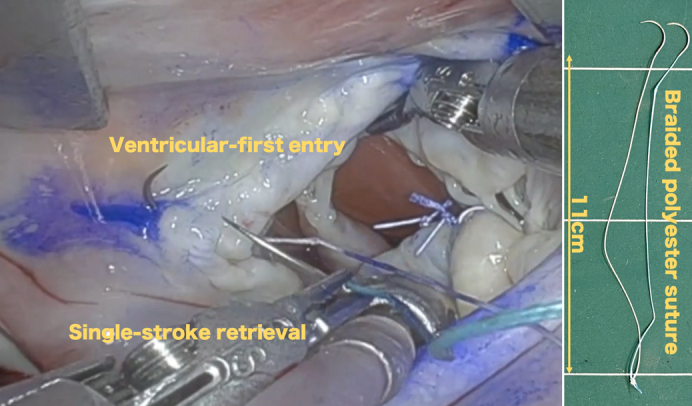
Robotic annuloplasty using 11-cm braided sutures and ventricular-first anchoring.

**Central Message**

Standardized 11-cm continuous braided suturing optimizes robotic motion and stability, achieving a 0.4% ring dehiscence rate in 1224 cases.

Robotic technology has expanded the scope of minimally invasive mitral valve repair.^1^ However, annuloplasty remains one of the most technically demanding steps as the result of restricted access and the need for precise suture management. This article describes the evolution of our technique from interrupted to a standardized continuous suturing method, emphasizing practical details that ensure reproducibility and surgical efficiency.

## Surgical Setup

Robotic mitral valve repair is performed via a right thoracic approach using a standard robotic platform. After the establishment of cardiopulmonary bypass, the mitral valve—and when indicated, the tricuspid valve—is sequentially exposed. Leaflet repair is completed before annuloplasty. Details regarding patient positioning and port placement have been described previously. Institutional review board approval was not required for this study because it is a description of a surgical technique and does not involve original clinical research data or patient-identifiable information. All patients provided informed consent for the use of their intraoperative images and videos for educational and publication purposes.

## Evolution of Annuloplasty Technique

### Phase 1: Interrupted Sutures

During the initial phase of robotic mitral valve repair, annuloplasty was performed using interrupted sutures. This approach allowed precise control of each stitch and provided reliable fixation; however, frequent needle exchanges and suture handling resulted in prolonged aortic crossclamp times.

### Phase 2: Interrupted Sutures Using Double-Arm U-Clips

To reduce the time and effort required for knot tying, interrupted annuloplasty using double-arm U-clips was adopted.^2^ This technique improved procedural efficiency; however, after the discontinuation of U-clip production and its distribution, this approach could no longer be continued.

### Phase 3: Transition to Continuous Suturing

With increasing experience, a hybrid approach combining continuous suturing with interrupted sutures at selected locations, such as the commissures, was introduced. When conventional monofilament running sutures were used, loosening of the suture during annuloplasty occasionally occurred, raising concerns regarding annular detachment from surrounding tissue. To address this issue, continuous annuloplasty using barbed sutures was developed.^3^ This method effectively prevented suture loosening and eliminated the need for knot tying at the beginning and end of the suture line. However, a specific limitation of barbed sutures was the risk of excessive annular constriction caused by overtightening, particularly in the robotic setting, where tactile feedback is limited.

### Phase 4: Current Standard Technique—Continuous Braided Suturing

#### Full-ring annuloplasty

**Figure 1:**
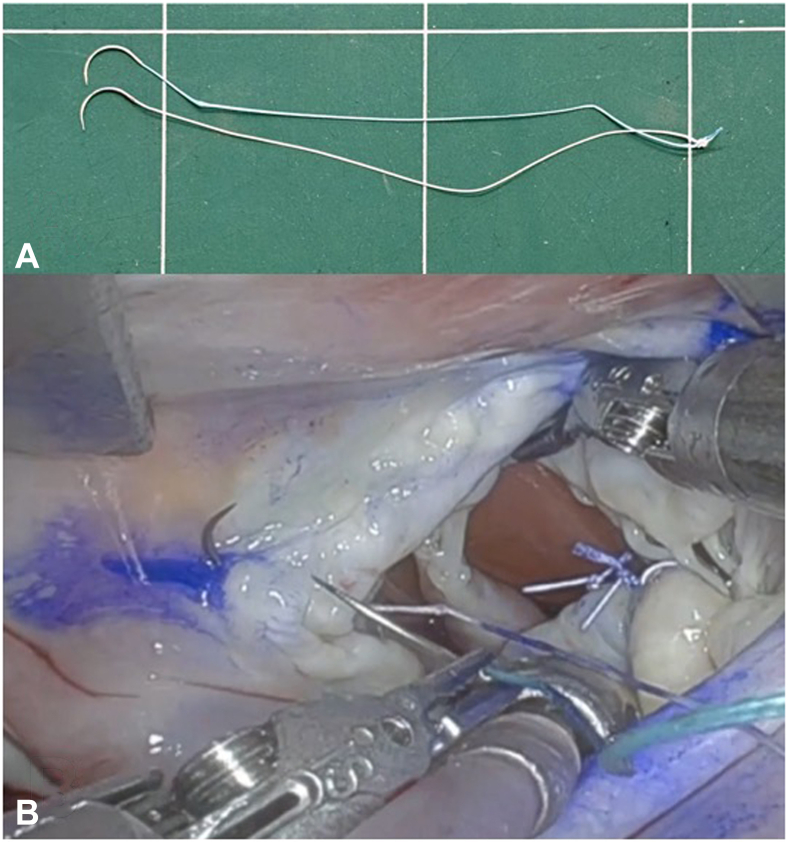
Full-ring annuloplasty: (A) two 11-cm sutures tied to create a 22-cm double-arm braided suture; and (B) “ventricular-first” stitch near the trigone.

We use double-armed 2-0 braided polyester sutures (ETHIBOND; Ethicon) (Video 1). Two sutures are tied together 11 cm from the needle to create a 22-cm double-arm suture (Figure 1, *A*). The initial stitch is placed from the ventricular side to the atrial side near the trigone (Figure 1, *B*). This "ventricular-first" entry ensures secure transmural purchase. The suture is advanced along the posterior annulus with consistent depth and pitch, typically completed in 10 minutes.

#### Partial-band annuloplasty

**Figure 2:**
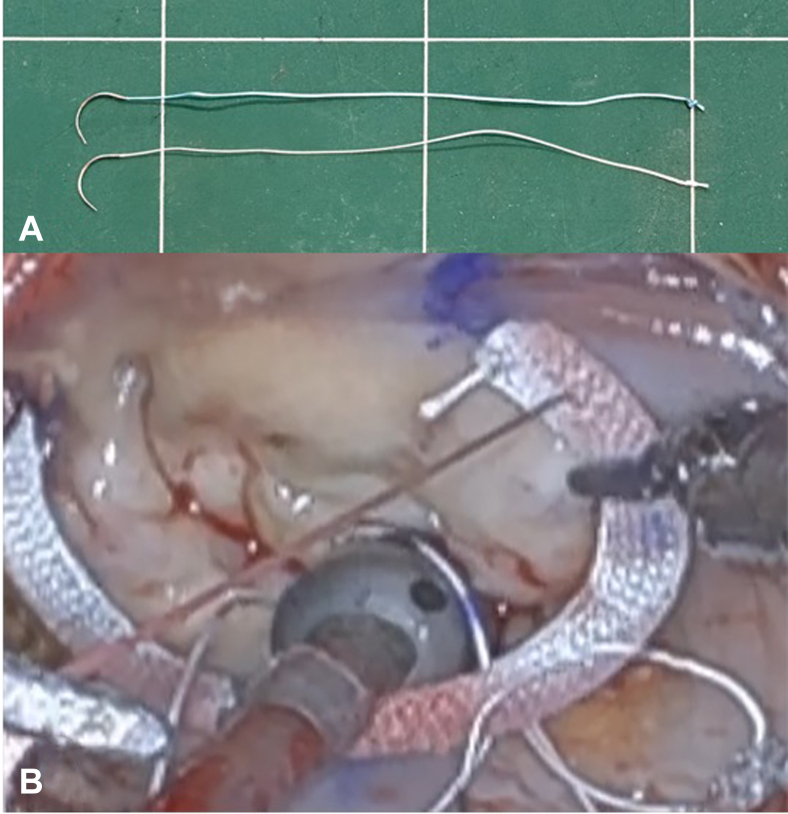
Partial-band annuloplasty: (A) 11-cm suture with a stopper knot and (B) direct initiation from the band.

For partial bands, a single 11-cm 2-0 ETHIBOND suture with a stopper knot is used (Figure 2, *A*). Suturing starts directly from the band without the ventricular-side entry (Figure 2, *B*). The stopper knot functions as an anchor. This approach is also applied to robotic tricuspid annuloplasty.

## Technical Pearls and Pitfalls

• Initiating from the left ventricular side ensures stable anchoring.
• Care must be taken to avoid entangling the chordae tendineae during the first stitch.
• Maintaining tension on the previous stitches prevents loosening.
• Preoperative marking of the trigones and P2 center assists in proper ring alignment.
• Take smaller bites on the prosthetic ring/band and larger bites on the native annulus to ensure a secure fit and proper reduction

## Discussion

Continuous annuloplasty is ideal for the robotic environment, where minimizing instrument exchanges and optimizing procedural flow are paramount. By standardizing the suture length to approximately 11 cm, we achieve “single-stroke” handling that allows the surgeon to pull the suture through in one fluid motion, significantly reducing crossclamp time. Although automated fastening devices (eg, Cor-Knot; LSI Solutions) are popular for interrupted sutures, their repetitive insertion is time-consuming and incurs significant cumulative costs. Our technique minimizes fastening points, offering a more cost-effective and efficient alternative without compromising the security of the repair.

At our institution, a semirigid complete annuloplasty ring (Memo 4D; Corcym) is routinely used as the primary device for mitral valve repair. In cases with a high anticipated risk of systolic anterior motion, a rigid partial band (Physio Flex; Edwards Lifesciences) is selected on the basis of the patient's anatomy. The exceptional stability of our results is attributed to the use of braided sutures, which allow for fine-tuned tension control and better pliability compared to monofilament sutures. Furthermore, the “ventricular-first” entry near the fibrous trigones ensures a robust transmural purchase, providing secure anchoring even in the absence of the tactile feedback typically available in open surgery.

This study is limited by its single-center, technical focus. Although clinical durability remains a critical factor in mitral valve repair, the primary objective of this report is to provide a reproducible and efficient protocol for robotic annuloplasty. Further longitudinal studies with long-term echocardiographic follow-up are warranted to confirm the long-term surgical outcomes. The transition from interrupted to this continuous method represents a practical evolution that balances surgical efficiency with anatomical precision. This technique is easily adaptable to tricuspid annuloplasty and can facilitate the safe adoption of robotic cardiac surgery for teams in their early learning curve.

## Conclusions

Annuloplasty in robotic mitral valve repair can be simplified and standardized using continuous suturing techniques. This approach enables secure fixation within a short operative time and can be readily applied to robotic tricuspid annuloplasty.

## Conflict of Interest Statement

The authors reported no conflicts of interest.

The *Journal* policy requires editors and reviewers to disclose conflicts of interest and to decline handling or reviewing manuscripts for which they may have a conflict of interest. The editors and reviewers of this article have no conflicts of interest.

## Supplementary Data

Video 1 Standardized continuous braided suturing technique for robotic mitral valve annuloplasty. Video available at: https://www.jtcvs.org/article/S2666-2507(26)00128-8/fulltext.
