# Supplementary figures and images for: Continuous braided suturing technique for robotic mitral valve annuloplasty
Source: JTCVS Tech. 2026 Mar 20;37:102321. doi: 10.1016/j.xjtc.2026.102321 (PMC13261163; doi:10.1016/j.xjtc.2026.102321)

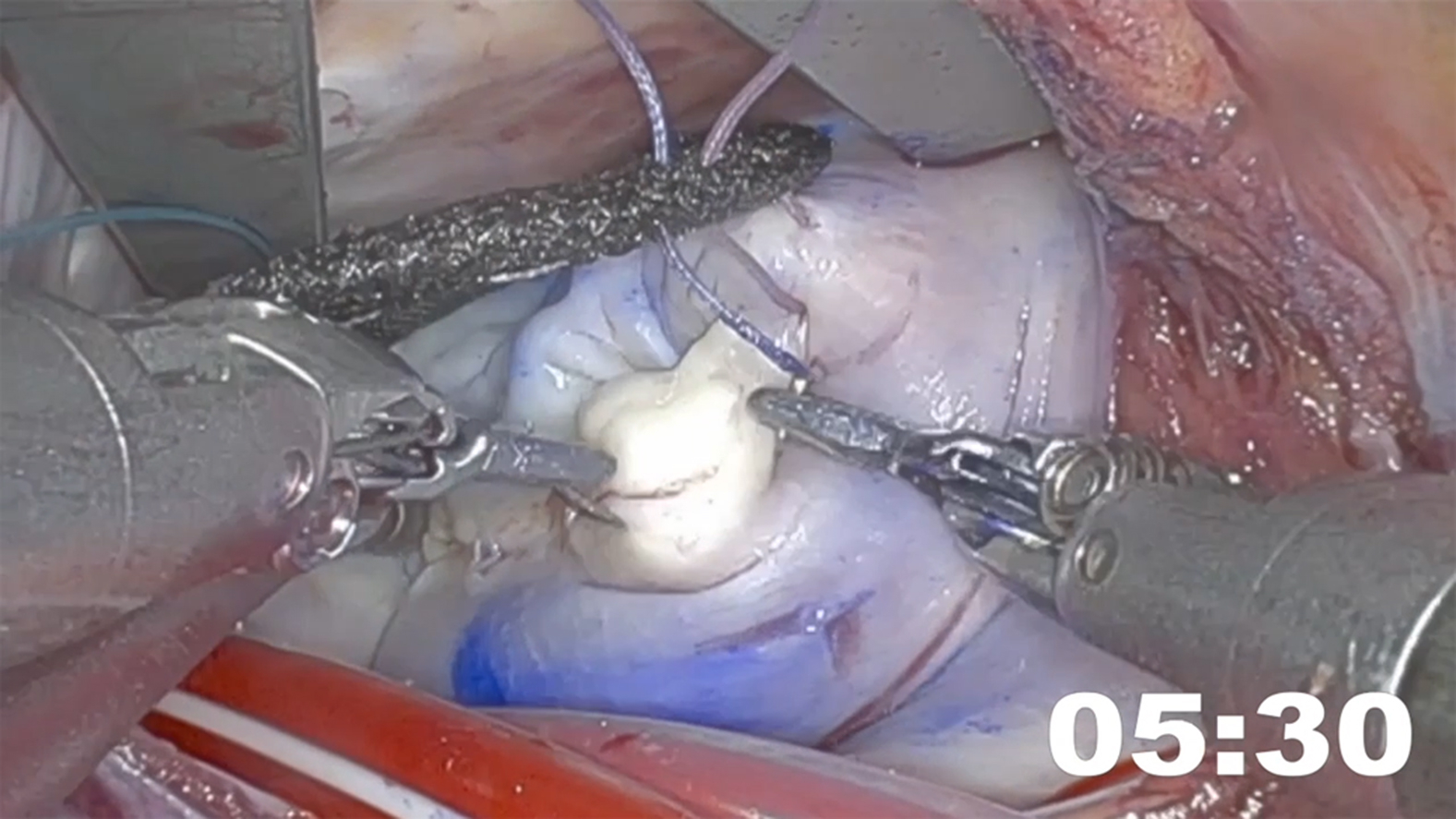

Supplement: Video 1 — Standardized continuous braided suturing technique for robotic mitral valve annuloplasty. Video available at: https://www.jtcvs.org/article/S2666-2507(26)00128-8/fulltext. [file fx2.jpg]
